# COVID-19: An Update about the Discovery Clinical Trial

**DOI:** 10.3390/ph13050098

**Published:** 2020-05-14

**Authors:** Jean Jacques Vanden Eynde

**Affiliations:** Formerly head of the Department of Organic Chemistry (FS), University of Mons-UMONS, 7000 Mons, Belgium; jean-jacques.vandeneynde@ex.umons.ac.be

**Keywords:** chloroquine, COVID-19, hydroxychloroquine, lopinavir/ritonavir, plasma, remdesivir, SARS-CoV-2

## Abstract

Finding efficacious and safe treatments for COVID-19 emerges as a crucial need in order to control the spread of the pandemic. Whereas plasma therapy attracts much interest, the European project Discovery focuses on the potentialities of small molecules like remdesivir, the combination of lopinavir/ritonavir, hydroxychloroquine, and chloroquine. Results recently published on the clinical evaluation of those drugs are compiled in this brief report, although complete data are still impatiently awaited.

## 1. Introduction

Deconfinement linked to COVID-19 is becoming a huge cause of concern for inhabitants of many countries and their political leaders. In the meantime, researchers from diverse scientific fields are gathering their efforts in order to combat SARS-CoV-2. Targeting the virus itself, testing infected people, offering treatments, seeking immunized patients, and developing vaccines are among the numerous avenues actually considered. On March 13, the World Health Organization (WHO), in collaboration with several partners, created the SOLIDARITY Response Fund to support studies on COVID-19 [1]. In the wake, the French Institut National de la Santé Et de la Recherche Médicale (INSERM) launched a European initiative in the form of a clinical trial named Discovery. A couple of weeks ago, we briefly reviewed the first potential treatments under study in those projects [2]. We now wish to provide an updated situation.

## 2. Current Clinical Trials: An Overview

As of May 4, the search term “COVID-19” in the database of the U.S. National Library of Medicine of the National Institutes of Health (Bethesda, Md, USA) [3] gave 1133 answers. Less than 6 weeks before (March 28), the same query had resulted in 202 titles (Table 1). That incredible increase clearly reflects the worldwide concern created by the disease.

**Figure 1:**
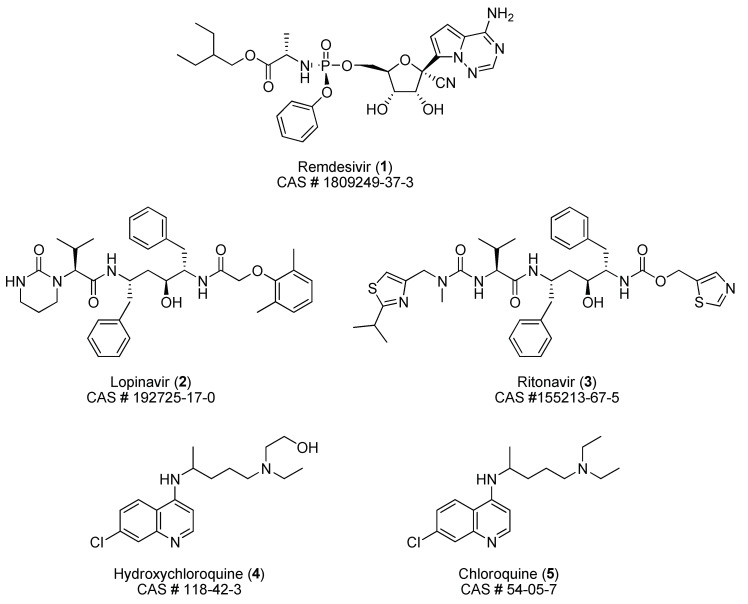
Structure of remdesivir (**1**), lopinavir (**2**), ritonavir (**3**), hydroxychloroquine (**4**), and chloroquine (**5**).

Considering the most cited options, as of March 28 [2], for a potential treatment, we analyzed the evolution of the number of clinical trials on April 8, April 28, and May 4. As indicated in Table 1, actually, researches on the efficacy of hydroxychloroquine (**4**, Figure 1) and plasma therapy constitute, by far, the most explored fields. They are followed by studies involving chloroquine (**5**) and the combination lopinavir (**2**)/ritonavir (**3**). To date, tocilizumab emerges as a lead in the category of monoclonal antibodies and cell therapy is focused on the potentialities of mesenchymal stem cells. Many other small molecules (essentially antiviral agents) and proteins remain attractive subjects of trials, but to a lesser extent.

Comparison with clinical trials reported in the Chinese Clinical Trial Registry [4] deserves some attention. In that registry, the vast majority of trials concern traditional Chinese medicine (plant extracts). Convalescent plasma and stem cells therapies generate an evident interest whereas most drugs and antibodies commercialized in the Western countries are absent of the studies. However, it is noteworthy that the usefulness of hydroxychloroquine, but above all that of chloroquine, is intensively investigated. Among the tested antiviral agents, mention should be made of the combination lopinavir/ritonavir, favipiravir (approved in Japan), and umifenovir (available in China and Russia).

## 3. The Discovery Clinical Trial

On March 22, the INSERM announced the start of a European adaptive clinical trial entitled “Trial of Treatments for COVID-19 in Hospitalized Adults (DisCoVeRy)” (NCT04315948). [5] The trial is supposed to enroll 3,100 patients in seven countries, namely France, Spain, the United Kingdom, Germany, Luxemburg, the Netherlands, and Belgium. [6] It started in three French hospitals: Centre Hospitalier Régional Universitaire de Lille (Lille, France), Centre Hospitalier Universitaire de Nantes (Nantes, France), and Assistance Publique Hôpitaux de Paris—Bichat Claude Bernard (Paris, France). To date, there are 34 study hospitals, 32 in France and 2 in Luxembourg. The five other countries are not yet represented [7].

Initially, the drugs to be evaluated in the Discovery project are

- remdesivir (**1**);
- the combination lopinavir (**2**)/ritonavir (**3**), eventually with the addition of interferon β-1a;
- hydroxychloroquine (**4**) or chloroquine (**5**), eventually associated with an antibiotic (azithromycin or other).

As indicated in the protocol, exclusion criteria comprise, among others,

- Patients with liver damage as indicated by alanine/aspartate aminotransferases levels, in blood, more than 5 times the upper limit of normal;
- Patients suffering of severe chronic kidney disease;
- Patients using medications that are contraindicated with lopinavir/ritonavir (e.g., amiodarone, colchicine, simvastatine) or hydroxychloroquine (e.g., citalopram, escitalopram, hydroxyzine, domperidone, piperaquine).

It is noteworthy that suffering of cardiac problems was not a criterion of exclusion from the study.

The estimated study completion date has been fixed on March 2023, 3 years after the starting date. Meanwhile, some partial reports on the efficacy of those molecules in the treatment of COVID-19 have been made public. Their conclusions are often contradictory as reported hereafter.

### 3.1. Remdesivir (**1**)

Remdesivir (GS-5734™) is an antiviral agent developed by Gilead Sciences in the frame of the Ebola virus outbreak. It was considered in one clinical trial only on the subject: “Investigational Therapeutics for the Treatment of People with Ebola Virus Disease” (NCT03719586). [8] The study was interrupted because the number of deaths in the group receiving remdesivir was higher than in the control group [9]. The molecule was designated as an orphan drug for treatment of Ebola disease on September 18, 2015 [10]. Gilead was also granted the orphan drug designation for treatment of COVID-19 in March 23, 2020. [11] However, 2 days later, under an explosion of criticisms, the company asked for a cancellation of its designation [12]. Access to the drug has also been expanded, exceptionally, to the US army [13].

The first patient infected by SARS-CoV-2 in the United States was hospitalized on January 19 and treated with compassionate-use remdesivir. [14] The authors concluded: “… As of January 30, 2020, the patient remains hospitalized. He is afebrile, and all symptoms have resolved with the exception of his cough, which is decreasing in severity. … Although a decision to administer remdesivir for compassionate use was based on the case patient’s worsening clinical status, randomized controlled trials are needed to determine the safety and efficacy of remdesivir and any other investigational agents for treatment of patients with 2019-nCoV infection.”

At the end of January, Gilead accepted to provide remdesivir for compassionate-use to a series of clinicians in Austria, Canada, France, Germany, Italy, Japan, the Netherlands, Spain, and the United States. The authors of the study concluded that “In this cohort of (53) patients hospitalized for severe Covid-19, clinical improvement was observed in 36 of 53 patients (68%). Measurement of efficacy will require ongoing randomized, placebo-controlled trials of remdesivir therapy.” [15] 

Gilead has extended the research (NCT04323761 [16]) by providing intravenous infusions of remdesivir to 155 hospitals in 12 countries: Belgium (4 hospitals), Canada (1), France (9), Germany (7), Israel (3),Italy (7), Netherlands (2), Romania (1), Spain (10), Switzerland (6), the United Kingdom (4), and the United States (101).

In a press release, on April 16, K. Mullane, from the university of Chicago announced that 125 patients, mostly with severe COVID-19, had entered into the phase 3 trial and she concluded that “the best news is that most of our patients have already been discharged, which is great. We’ve only had two patients perish.” However, no placebo group was included in the trial. [17]

On April 29, Gilead disclosed another partial result concerning the efficacy of 5-day and 10-day treatments among 397 patients distributed in a 1:1 ratio (200:197). Results indicated that “the study demonstrated that patients receiving a 10-day treatment course of remdesivir achieved similar improvement in clinical status compared with those taking a 5-day treatment course.” A. Subramanian, a lead investigator for the Stanford University School of Medicine, added: “While additional data are still needed, these results help to bring a clearer understanding of how treatment with remdesivir may be optimized, if proven safe and effective.” [18]

In an independent study, Wang *et al*. examined 236 patients over 10 hospitals in the province of Hubei, China. In this randomized, double-blind, placebo-controlled trial, 158 people were administered remdesivir and 78 received a placebo. The authors interpreted their results in the following terms: “In this study of adult patients admitted to hospital for severe COVID-19, remdesivir was not associated with statistically significant clinical benefits. However, the numerical reduction in time to clinical improvement in those treated earlier requires confirmation in larger studies.” [19].

**Figure 2:**
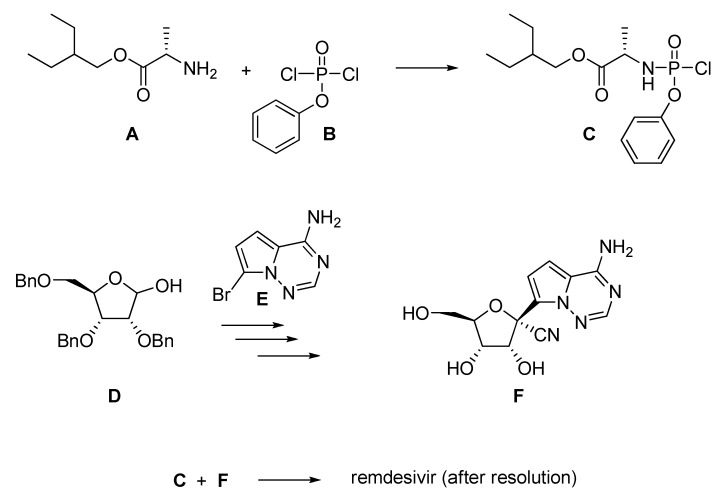
Schematic synthesis of remdesivir (**1**). Yields were not mentioned in [20].

From a chemical point of view, it must be underlined that remdesivir has six chiral centers and is manufactured as a single stereoisomer under aseptic conditions. Globally, its synthesis requires several key intermediates (**A**–**F**) represented in Figure 2 [20]. Therefore, production relies on different suppliers whose capacities must be expanded in order to satisfy the eventual high demand of doses, as underlined by Gilead [21,22]. 

### 3.2. The Combination Lopinavir/Ritonavir (**2**/**3**)

Commercially available under the brand name Keletra^®^, the combination is manufactured by AbbVie in several countries of the world, sometimes in a generic version. Availability is not a concern.

The combination has been the subject of more than 400 registered clinical trials [3] and it is prescribed for treatment of HIV infection.

In the case of COVID-19, the combination lopinavir/ritonavir is evaluated in 49 clinical trials. There are several anecdotal reports indicating that Japanese, Chinese, and Taiwanese patients treated with lopinavir/ritonavir survived the disease, but nothing indicated the benefit of the use of those antiviral agents. Addition of umifenovir (Arbidol^®^) to the combination did not show any improvement in the treatment [23,24,25,26,27]. A randomized, controlled, open-label trial conducted in Wuhan (China) concerned two groups of 99 and 100 patients, respectively. The first group was assigned the combination of antiviral drugs whereas the second group received standard care. The authors [28] concluded: “we found that lopinavir–ritonavir treatment did not significantly accelerate clinical improvement, reduce mortality, or diminish throat viral RNA detectability in patients with serious Covid-19.”.

### 3.3. Hydroxychloroquine (**4**) and Chloroquine (**5**), Eventually with the Addition of an Antibiotic

Both molecules are frequently prescribed for the treatment of malaria since decades and have been largely studied. Their side-effects are widely known as well as all their pharmacological properties. Both hydroxychloroquine (Plaquenil^®^) and chloroquine (Aralen^®^) have been clinically tested for their anti-HIV activity [3,29]. Hydroxychloroquine is also recommended to treat lupus erythematosus and rheumatoid arthritis [30].

Hydroxychloroquine and chloroquine, eventually associated with an antibiotic, are the subjects of many contradictory reports about their usefulness in the treatment of COVID-19. Positive effects with viral clearance have been observed whereas other groups concluded at the absence of efficacy or at cardiotoxicity when compared with standard care. Discussions are articulated among four main aspects that should be taken in account: (i) stage of the disease; (ii) presence/absence of a control group; (iii) efficacy; (iv) safety.

As earlier mentioned, controversial results have been published on the efficacy of treatments associating hydroxychloroquine and azithromycin for French patients hospitalized for moderate [31] or severe [32,33] COVID-19 infection. Another trial [34], involving male patients only at US Veterans Health Administration medical centers, suggested caution in using hydroxychloroquine, particularly when not combined with azithromycin.

Regarding cardiotoxicity, it was attributed either to the combination of hydroxychloroquine and azithromycin or to each of those two drugs independently. Thus, a study [35] indicated that, for patients treated with the combination, critical corrected QT interval prolongations were higher than for patients treated with the antibiotic alone. This contrasts with the conclusion of J.C.E. Lane *et al*. [36] who reported that “short-term hydroxychloroquine treatment is safe, but addition of azithromycin may induce heart failure and cardiovascular mortality, potentially due to synergistic effects on QT length.” On the other hand, data [37] collected from four French hospitals revealed that patients receiving hydroxychloroquine experienced electrocardiogram modifications requiring discontinuation of the treatment with that molecule.

In the case of chloroquine, studies on dose-dependence safety can be found. A Brazilian trial [38] revealed that treatment had to be interrupted for a cohort of patients allocated to receive a total dose of chloroquine of 12 g, 4-fold the dosage recommended in the Discovery project. The results were confirmed in a French study [39] that concluded that “high-dose chloroquine treatment regimens which result in whole blood chloroquine concentrations below 10µmol/L for the majority of patients should not result in life-threatening cardiovascular toxicity.”

All those observations confirm a recent theoretical model that attracted attention to the fact that both hydroxychloroquine and chloroquine were characterized by narrow therapeutic windows overlapping with the highest tolerated doses [40]. The problem could be overcome by using the medications as an aerosol as suggested by Klimke et al. [41].

## 4. Conclusion

As of May 4, there are 1133 clinical trials on COVID-19 that are referenced in the U.S. National Library of Medicine of the National Institutes of Health [3]. The most considered potential treatments are centered on plasma therapy and evaluation of the potentialities of hydroxychloroquine, chloroquine, and the combination lopinavir/ritonavir. Protein and cell therapies attract less interest, despite their high promises in other diseases. The antiviral agent remdesivir exerts a moderate focus in the medical community, despite a large mediatic audience.

Great hope had been put in the European trial Discovery. It must be admitted, however, that the actual situation is rather disappointing since many countries are not yet enrolled and consequently not recruiting. Results on the efficacy of remdesivir are fragmentary and disclosed by the manufacturer. The combination lopinavir/ritonavir does not provide promising curative effects. In-depth studies are still awaited in order to clarify contradictory data on the usefulness of hydroxychloroquine and chloroquine. In conclusion, to date no treatment can be proposed despite the urgency of the situation.

## Figures and Tables

**Table 1:** Most cited potential treatments for COVID-19 following the U.S. National Library of Medicine [3] and the Chinese Clinical Trial Registry [4].

Search term	Number of clinical trials				
	Following [3]				Following [4]
	As of 03/28	As of 04/08	As of 04/28	As of 05/04	As of 05/04
COVID-19	202	366	997	1133	631
Hydroxychloroquine	19	58	148	165	12
Plasma	na	na	123	135	13
Chloroquine	12	23	52	55	26
Lopinavir (+ ritonavir)	14	22	44	49	9
Tocilizumab	6	15	32	35	3
Mesenchymal (cells)	na	14	30	30	16
Remdesivir	9	9	18	19	0
Oseltamivir	4	6	12	15	0
Methylprednisolone	5	6	14	14	0
Favipiravir	2	4	12	13	6
Sarilumab	4	5	12	12	0
Umifenovir *	9	10	11	11	2
Losartan	2	5	10	10	0
Baricitinib	2	2	9	10	0
Colchicine	2	4	8	9	0
Bevacizumab	2	2	3	3	0
Thalidomide	2	2	3	3	0

* including 3 studies on Abidol; nd = not analyzed on that date.
